# Neuroprotective effects of telmisartan in a harmaline-induced model of essential tremor: modulation of the renin-angiotensin system and inflammatory pathways

**DOI:** 10.3389/fphar.2026.1810499

**Published:** 2026-05-26

**Authors:** Sama M. Farrag, Amr M. Emam, Ahmed S. Kamel, Muhammed A. Saad, Mona A. Kortam, Noha H. Sayed, Nevine Fathy

**Affiliations:** 1 Department of Pharmacology and Toxicology, Faculty of Pharmacy, Cairo University, Cairo, Egypt; 2 Department of Pharmacology and Toxicology, College of Pharmaceutical Sciences and Drug Manufacturing, Misr University for Science and Technology (MUST), 6th of October City, Giza, Egypt; 3 Department of Pharmacology and Toxicology, Faculty of Pharmacy and Drug Technology, Egyptian Chinese University, Cairo, Egypt; 4 Department of Pharmaceutical Sciences, College of Pharmacy, Gulf Medical University, Ajman, United Arab Emirates; 5 Department of Biochemistry, Faculty of Pharmacy, Cairo University, Cairo, Egypt

**Keywords:** angiotensin receptors, AT1 and AT2 receptors, bergmann cells, essential tremor, inflammatory cytokines, mas receptor, neurodegeneration, telmisartan

## Abstract

**Background:**

Essential tremor (ET) is a widespread neurological disorder with mild cerebellar degeneration that affects motor coordination, causing involuntary, rhythmic tremors during action. Excessive brain renin-angiotensin system activation favors pro-inflammatory milieu that is associated with neurodegenerative diseases.

**Objective:**

The present study aimed to investigate the neuroprotective effects of Telmisartan (TELMI), an angiotensin receptor blocker, in a harmaline-induced model of essential tremor.

**Methods:**

Male rats were assigned to three groups: control, harmaline (30 mg/kg; i. p.) and a treatment group receiving TELMI (3 mg/kg/day; p. o.) administered 5 days before harmaline induction.

**Results:**

TELMI reduced tremor severity and enhanced performance in the rotarod, wire grip strength, footprint test, open field test, and gait kinematics. Cerebellar molecular analysis revealed that TELMI significantly increased MAS receptor expression and angiotensin 1-7 levels, while concurrently downregulating angiotensin II type 1 and 2 receptors. The levels of ACE1/ACE2 confirmed the favoring of the beneficial RAS arm. This modulation notably inhibited the activation of p38 MAPK and reduced pro-inflammatory cytokines, including TNF-alpha, IL-1-beta, IL-6, and chemokine receptor type 4. Neurotransmitter analysis showed restoration of the excitatory/inhibitory balance between GABA and glutamate levels. Immunohistochemical analysis of Bergmann cells in the cerebellum demonstrated marked reductions in the proliferative marker; Ki67, and markers of glial and progenitor cell activity; S100β and SOX2 expression, respectively, concurrently with, the suppression of the neural stem cell marker; nestin.

**Conclusion:**

These findings suggest that TELMI protects against cerebellar neurodegeneration in essential tremor by exerting anti-inflammatory, anti-excitotoxic, and receptor-modulatory effects through the Ang 1–7/MAS receptor and p38 MAPK signaling pathways.

## Graphical Abstract

**Figure:**
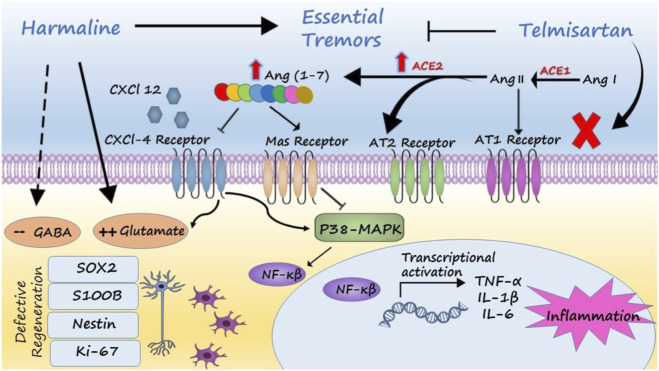
Neuroprotective effects of Telmisartan in a harmaline-induced model of essential tremor via targeting ACE2/Ang 1–7/MASR axis. ACE1, angiotensin converting enzyme 1; ACE2, angiotensin converting enzyme 2; Ang 1–7, angiotensin 1–7; AT1, angiotensin II type 1 receptor; AT2, angiotensin II type 2 receptor; CXCR4, chemokine receptor type 4; CXCL12, CXC motif chemokine 12; GABA, gamma aminobutyric acid; IL-1β, interleukin-1 beta; IL-6, interleukin-6; Ki67, antigen Kiel 67; NF-κB, nuclear factor kappa B; p38 MAPK, p38 mitogen-activated protein kinase; S100β, S100 calcium binding protein B; SOX2, SRY-box transcription factor 2.

## 1 Introduction

Tremors are common movement disorders marked by involuntary rhythmic and oscillatory movement of a body part, typically maintaining a consistent frequency but with varying amplitude. Essential tremor (ET) is the leading neurological cause of postural or action tremors. It usually manifests as a bilateral postural tremor of the hands, but as the condition progresses, it can also affect the head and voice (and less frequently, the legs and trunk) (Bhatia et al., 2018). Despite its benign effect on life expectancy, ET symptoms are progressive and potentially debilitating, often forcing the patient towards social withdrawal and early retirement (Lorenz et al., 2006). Worthy of note, ET is believed to be a risk factor for the development of Parkinson’s disease (PD) (Tarakad and Jankovic, 2019).

The etiology of ET is largely unknown. Nearly 50% of the cases were reported to stem from a genetic mutation, although no particular gene has been pinpointed. The varying age of onset, the presence of sporadic cases, and incomplete concordance of ET among monozygotic twins indicate that environmental triggers are also entangled. ET is estimated to affect up to 1% of the global population, with family history composing nearly half of the cases (Louis, 2018). Although the incidence increases with age, familial background often impacts younger individuals (Song et al., 2021).

Clinically, neurodegenerative changes are most evident in the cerebellum of ET patients with a specific loss of Purkinje cells (PCs) along with signs of swelling and degeneration of their axons. Furthermore, the dentate nucleus in ET exhibits loss of the gamma aminobutyric acid (GABA) receptors that is directly associated with the duration of the disease (Louis et al., 2007).

Multiple experimental models have been developed to replicate ET, among which the harmaline (HRL)-induced model demonstrated the highest translational relevance. It has gained cogent recognition for providing empirical insights into ET pathogenesis in preclinical studies (Handforth, 2012).

The first-line ET therapies, whether it is the beta blocker, propranolol and/or the anticonvulsant, primidone, come with several contraindications and undesirable side effects that limit their use. Lately, invasive procedures and botulinum toxin are being employed. Notwithstanding, all these therapies are for the management of symptoms, and none of which offers a cure for ET (Kumar Pal, 2011).

Renin-angiotensin system (RAS) might have a crucial role in the regulation of neurological functions by influencing glutamate (GLU)/GABA release in the brain. To add, activation of the angiotensin II type 1 receptor (AT1) is believed to promote neuroinflammation via triggering a cascade of pro-inflammatory responses, including the upregulation of cytokines, chemokines, and oxidative stress mediators, which contribute to neuronal damage and disease progression (Tsuda, 2012; Benicky et al., 2011). AT1 activation was reported to have amyloidogenic activity, ensuing amyloid beta (Aβ) deposition—a defining feature of Alzheimer’s disease (AD) pathology (Abdelkader et al., 2020). In PD, AT1 activation has been linked to enhanced alpha-synuclein aggregation and dopaminergic neuron degeneration, indicating a direct role in neurodegenerative pathology (Lage et al., 2024). Moreover, AT1-mediated signaling was found to aggravate microglial activation and astrocytic dysfunction, further amplifying inflammatory responses within the CNS (Rao et al., 2024). Thus, therapeutic blockade of AT1 receptors holds significant potential for alleviating neuroinflammation and offering neuroprotection in neurological disease contexts.

Telmisartan (TELMI), a widely used anti-hypertensive medication, is a nonpeptide AT1 antagonist, as well as, an activator for peroxisome proliferator-activated receptor γ (PPARγ) (Liu et al., 2020). Beyond its role in managing hypertension, TELMI has demonstrated neuroprotective potential. It can mitigate cognitive and executive impairments, neuroinflammation and oxidative stress, neuronal and synaptic injury, as well as Aβ pathology in AD (Fu et al., 2023). Further, TELMI offered protection against epileptic seizures invoked by cytokines (Pushpa et al., 2014). These merits were mostly attributed to its capability to penetrate the blood-brain barrier, inhibiting centrally mediated effects of angiotensin II, the pressor arm of RAS, while enhancing the production of angiotensin 1–7 (Ang 1–7), which activates the depressor, protective arm (Gohlke et al., 2001). Ang 1–7 has demonstrated significant neuroprotective effects in various neurological conditions. By binding to Mas receptors (MASR), Ang 1-7 was found to promote anti-inflammatory, antioxidant, and vasodilatory responses, and hence reduces oxidative stress and inflammation, which are critical contributors to neurodegenerative diseases and cognitive decline (Rukavina Mikusic et al., 2021). Additionally, Ang 1–7 has been shown to enhance neuronal survival and to improve vascular health, making it a promising therapeutic target for several neurocognitive disorders (Lucas et al., 2025).

Although TELMI has been reported to possess neuroprotective properties in different neurological disorders, its potential role in ET has not been adequately explored. To the best of our knowledge, this is the first study sought to evaluate the neuroprotective effect of TELMI against ET in HRL rat model, and to assess its prospect to modulate GLU/GABA balance as well as cytokines’ release. The study further explored the cerebellar S100β/SOX2/p38 MAPK as a key signaling pathway involved in TELMI-ensued protective role.

## 2 Materials and methods

### 2.1 Animals

*Wistar* male rats (8–10 weeks) weighing 200–250 g were purchased from the animal facility of Egyptian drug authority (Giza, Egypt). Before conducting the experiment, animals were acclimatized for 7 days at the animal facility at the Faculty of Pharmacy, Cairo University (Cairo, Egypt). The experimental and adaptation conditions were controlled, with room temperature (25 °C ± 2 °C), 12 h dark and 12 h light cycles and humidity (60% ± 10%). Food and water were accessed *ad libitum*. All experimental procedures were revised and carried out with the Ethics Research Committee, Faculty of Pharmacy, Cairo University (BC3719) in strict application of the international policies of the US National Institute of Health for laboratory animals care (NIH Publications No. 85–23, updated 2011). All endeavors to lower animal suffering were taken. The sample size was calculated by the G-power calculator (V3.1, City Düsseldorf, Germany) using power (1- *β* = 0.8) besides the following measures; effect size η2 = 0.8, α-level = 0.05.

### 2.2 Drugs

Harmaline HCl dihydrate (HRL) was obtained from Sigma-Aldrich (Merck KGaA, Darmstadt, Germany). TELMI was obtained from Utopia pharmaceuticals company (Cairo, Egypt). HRL in saline (0.9% NaCl) vehicle was injected intraperitoneally (i.p.). TELMI was dissolved in 2% dimethyl sulfoxide (DMSO) and then completed with distilled water to the final volume for oral administration (p.o.). All chemicals and reagents (analytical grade) utilized in this study were obtained from Sigma-Aldrich (Merck KGaA, Darmstadt, Germany).

### 2.3 Harmaline-induced essential tremor

Harmaline is a well-established acute prescreening model for ET, commonly administered as a single dose. The success of the model induction is defined by the appearance of a characteristic, generalized action tremor involving limbs, trunk, and whiskers, which is most evident during movement and diminishes at rest. This phenotype represents the primary and most widely accepted functional marker of the model. In the present study, administration of harmaline (30 mg/kg, i. p.) produced a rapid-onset, sustained tremor that endured throughout the 3-hour-behavioural testing window, in line with previously established protocols demonstrating that this dose reliably induces stable tremor suitable for pharmacological and behavioural assessments (Handforth, 2012; Fatemeh Rahimi Shourmasti et al., 2014; Kamel et al., 2023; Shabani and Naderi, 2022). This acute protocol has been widely used to assess locomotor dysfunction and tremor-related changes (Abbassian et al., 2016; Abdulrahman et al., 2017; Kosmowska et al., 2017; Mohammadi et al., 2019; Akman et al., 2021). Generally, harmaline induces dose-dependent whole-body tremor in rats at doses ≥4 mg/kg, with 10–20 mg/kg commonly employed (Abdulrahman et al., 2017; Akman et al., 2021), while a single intraperitoneal dose of 30 mg/kg has been specifically validated to produce robust essential tremor-like symptoms (Handforth, 2012; Fatemeh Rahimi Shourmasti et al., 2014). Although repeated dosing paradigms have been explored, they may lead to tolerance and increased variability, whereas a single high dose provides a consistent and reproducible model, albeit with potential neurotoxic effects (Handforth, 2012). Multi-day or repeated-dosing protocols have been explored in the literature. While some have reported changes in tremor severity, they had similar behavioral outcomes; however, in several cases, comprehensive mechanistic and histopathological validation remains limited (Pirmoradi et al., 2025a; Pirmoradi et al., 2024; Pirmoradi et al., 2025b). Therefore, the acute single-dose approach used in this study aligns with established literature and provides a robust and reliable framework for investigating tremor mechanisms and evaluating potential therapeutic interventions. The successful establishment of the model was further supported by significantly elevated tremor scores, reflecting increased severity and duration of episodes, together with marked impairments in motor coordination and locomotor performance as evidenced by multiple behavioural paradigms, including open field, rotarod, wire grip, and footprint analyses. All the aforementioned functional deficits are indicative of cerebellar dysfunction, a key feature underlying ET pathophysiology.

### 2.4 Experimental design

**SCHEME 1:**
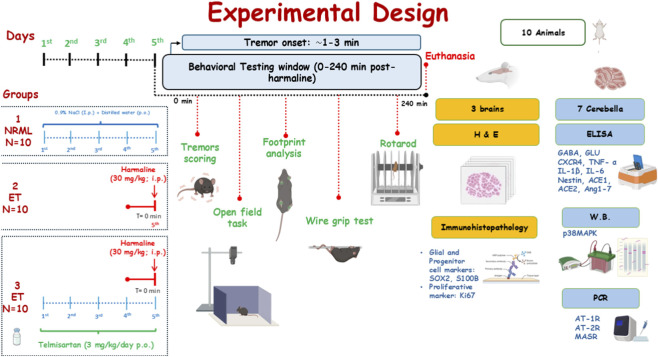
Experimental design of the study.

*Wistar* rats (n = 30) were randomly distributed blindly by a technical assistant into three different groups (n = 10/group) as presented in Scheme 1. The first group received saline solution (i.p.) and distilled water (p.o.) for five successive days. The second group was injected with HRL (30 mg/kg; i. p.) on the fifth day (Kamel et al., 2023) while the third group was treated with TELMI (3 mg/kg/day p. o.) for five consecutive days (Abdelkader et al., 2020) and afterwards was given HRL (30 mg/kg; i. p.) on the fifth day. The rats were assessed for ET within 3 h following harmaline injection using a tremor scoring system and neurobehavioral tests. In the present study, animals were deeply anesthetized with ketamine (100 mg/kg) and xylazine (10 mg/kg), administered intraperitoneally, followed by decapitation for rapid brain extraction. This approach was selected to facilitate prompt tissue collection and preserve the integrity of RNA and proteins required for downstream molecular analyses, including ELISA, qPCR, and Western blot. After euthanization, cerebella were isolated from the extracted brains on ice-cold plates by making two incisions and divided into two sets. The first set (n = 3) was allocated for histopathological examination after fixation in 10% neutral buffered formalin for 72 h. The second set (n = 7) was flash frozen in liquid nitrogen and divided into two equal portions. One part of the second set was used for biochemical analyses by ELISA technique, and the other part was analyzed by polymerase chain reaction (n = 3) and Western blot (n = 3). The samples were stored at−80 °C. The sample identity was masked during analysis, while coding and decoding of the samples were done through a separate technical investigator.

### 2.5 Methods

#### 2.5.1 Essential tremor evaluation

##### 2.5.1.1 Tremor scoring

After induction of ET, tremors’ severity was evaluated in all animals. The evaluation was rated by blinded observers based on latency until tremors appeared, duration of tremors until cessation, and degree of severity. The severity was scored quantitatively (Kamel et al., 2023); score 0 was given if there is no tremor, score 1 was recorded for mild tremor disseminates neck and head only, score 2 for moderate severity with intermittent tremors disseminates whole body = 2, score 3 for moderate but continuous tremors disseminates through whole body, score 4 was given for severe and constant tremor impedes locomotor function.

##### 2.5.1.2 Behavioral assessment

Motor behavioral tests were conducted with a 10-min Interval between every successive two tests with the following order: open field test, footprint analysis, wire grip then finally rotarod. The behavioral battery tests began with the exploratory test and ended by the most stressful test. All tasks were cleaned using 70% ethanol between trials.

##### 2.5.1.3 Open field test

The locomotor activity was evaluated in a plexiglass task measuring 90 cm wide by 90 cm long by 30 cm high. The interior of the arena was virtually divided equally into 16 squares, with central and peripheral areas. Horizontal exploration was quantified over 3 min Using video tracking ANY-maze software (Stoelting Co., USA) by recording the total distance travelled, speed, and immobility time (El-Kadi et al., 2025; Saad et al., 2025).

##### 2.5.1.4 Footprint test

The biomechanical function as well gait patterns in animals were measured by footprint test. A test was employed where rats’ hind paws were marked with nontoxic ink. The animals then traversed a transparent track of plexiglass [1 m length × 10 cm height × 10 cm width] supported in the floor with absorbent white sheets. Prior to testing, rats underwent three training sessions for habituation to the track. Footprint analysis was conducted by measuring the hind paws stride length and width. Stride length was recorded in the ipsilateral side and the study measured the distance between 2 consecutive footprints of the same hind paw. Stride width was defined as the length measured between 2 different paw prints in relation to the corresponding contralateral stride length (Abdelkader et al., 2020).

##### 2.5.1.5 Wire grip test

Muscle strength and motor balance were evaluated when animals grasp horizontally with their forepaws a fixed wire made of a steel (80 cm length, 7 mm diameter). The animals were subsequently suspended, and the duration they maintained their grip before falling was measured using a stopwatch (Murphy et al., 1995).

##### 2.5.1.6 Rotarod test

Ugo Basile rotarod apparatus (Model 47,750) was employed to evaluate motor coordination. This apparatus is formed of a non-slippery revolving rod with 3 cm diameter and 120 cm length compartmentalized by five disks to produce four equal sections, allowing for the testing of four rats concurrently. Animals underwent three daily training sessions of 5 min Each at a speed of 4 rpm for three successive days. During the test, the speed was automatically increased gradually from 4 to 40 rpm with a maximum duration of 5 min, and the time at which the rat remained on the rod before falling was recorded as fall-off time, detected by magnetic sensors located in trip boxes beneath the rod (Ibrahim et al., 2024).

#### 2.5.2 Evaluation of GABA, GLU, CXCR4, TNF-α, IL-1β, IL-6, nestin, ACE1, ACE2 and ang 1-7 by enzyme-linked immunosorbent assay (ELISA)

All assessments of parameters were performed using cerebella isolated from the extracted rat brains and done per the manufacturer’s instructions. Kits for GABA (Cat No # MBS045103, MyBiosource company, San Diego, USA), GLU (Cat No # MBS756400, MyBiosource company, San Diego, USA), interleukin-1 beta (IL-1β) (Cat No # ELR-IL1b-1, Ray Biotech, Inc., GA, USA), interleukin-6 (IL-6) (Cat No # ELR-IL6-1, Ray Biotech, Inc., GA, USA), tumor necrosis factor alpha (TNF-α) (Cat No # MBS9362408, MyBiosource, San Diego, CA, USA), nestin (Cat No # MBS064175, MyBiosource, San Diego, CA, USA), chemokine receptor type 4 (CXCR4) (Cat No # MBS3807610, MyBiosource, San Diego, CA, USA), angiotensin converting enzyme 1 (ACE1) (Cat No # MBS703086, MyBiosource, San Diego, CA, USA), angiotensin converting enzyme 2 (ACE2) (Cat No # MBS764117, MyBiosource, San Diego, CA, USA), and angiotensin 1–7 (Ang 1–7) (Cat No # MBS2022279, MyBiosource, San Diego, CA, USA) were used. The BCA kit supplied by G-Bioscience (USA) was used for assessing total protein concentrations in tissue. The study presented results as pg or ng/mg protein after dividing the assessed parameters’ concentrations (pg/ml or ng/ml) by the tissue protein content (mg/ml).

#### 2.5.3 Evaluation of p38 MAPK by western blot analysis

Cerebella isolated from the extracted rat brains were rinsed with phosphate buffer saline (PBS) and processed using protein extraction reagent; Ready Prep™ (Bio-Rad Inc., CA, USA), then protein concentration was determined by the Bradford assay. Mini-PROTEAN system (Bio-Rad) separated proteins on 10% sodium dodecyl sulfate (SDS)-PAGE gels using 120 V during 90 min. Then wet transfer apparatus (Bio-Rad) allowed proteins transfer to nitrocellulose membranes at 100 V for 1 h. For 1 h, the nitrocellulose was blocked at room temperature using 5% non-fat milk in TBST buffer. Primary antibody of p38 MAPK (Cat No # PA1 – 30,391, Thermo Scientific, Rockford, Illinois, USA), with dilution 1:1000 within the blocking buffer, was kept overnight incubated with the proteins at 4 °C. Then the membranes were washed and allowed to be incubated with horseradish peroxidase (HRP)-conjugated 2^ry^ antibodies at 1:5000 dilution in room temperature. After 1 h, TBST buffer was used for 10 min to wash membranes (3 times). The ECL detection system (Thermo Fisher Scientific) opted the proteins which were captured using a Bio-Rad ChemiDoc imaging system. The quantification of the bands’ intensities was performed using Bio-Rad Image Lab software and normalized relative to β-actin protein. The molecular weight of the detected proteins was highlighted using a protein marker (FastGene, NIPPON Genetics EUROPE GmbH).

#### 2.5.4 Real-time quantitative MAS, AT1, AT2 receptors

To estimate gene expression of cerebellar MAS, AT1, AT2 receptors, homogenization of cerebella isolated from the extracted rat brains was done in lysis buffer; TRIzol reagent, then total RNA was evaluated using RNeasy Mini kit (Qiagen, Venlo, Netherlands) and the degree of purity was assessed at wavelength 260/280 nm. Then qRT-PCR was done by SYBR Green JumpStart Taq ReadyMix (Sigma-Aldrich, St. Louis, MO, USA) after reverse transcription into complementary DNA using Reverse Transcription System (Promega, Leiden, Netherlands). The amplification was done using an Applied Biosystem StepOneTM (software version 3.1, California, USA). All procedures were in line to the guidelines of the respective manufacturers. The relative expression of assessed genes was calculated using the 2^−ΔΔCT^ formula with housekeeping gene; β-actin. The sequences of the primers are presented in Table 1.

**TABLE 1:** Primer sequence used for RT-PCR.

Parameter	Primer sequence (5′–3′)
*AT-1R*	F: CAG​CTT​GGT​GGT​GAT​TGT​C
	R: GCC​ATC​GGT​ATT​CCA​TAG​C
*AT-2R*	F: TAG​TCT​CTC​TCT​TGC​CTT​GG
	R: CTG​ACC​TTC​TTG​GAT​GCT​CT
*MASR*	F: ACT​GCC​GGG​CGG​TCA​TCA​TC
	R: GGT​GGA​GAA​AAG​CAA​GGA​GA
*β-actin*	F: CCC​ATC​TAT​GAG​GGT​TAC​GC
	R: TTT​AAT​GTC​ACG​CAC​GAT​TT

### 2.6 Histopathological evaluation

After flushing cerebellar tissue samples and fixing them in 10% neutral buffered formalin for 72 h, trimmed samples were treated in ascending grades of ethanol and cleared in Xylene. Infiltrated samples were embedded into Paraplast tissue embedding media. Rotatory microtome cut cerebella with 5 μm thick tissue sections to demonstrate cerebellar folia layers in different samples. The sections were stained by Hematoxylin and Eosin (H&E) and examined blindly by an experienced histologist using light microscope. All standard procedures, starting from samples’ fixation and staining, were according to Culling, C.F.A. 2013 (Culling, 2014).

### 2.7 Immunohistochemistry

After preparing 5-micron-thick tissue sections embedded in paraffin, immunohistochemical processing was performed using cerebella isolated from the extracted rat brains following the manufacturer’s protocol. The deparaffinized tissue sections were exposed for 20 min to 3% H_2_O_2,_ then incubated with SRY-box transcription factor 2 (SOX2) antibody (Cat No # EPR3131- Abcam, 1:100), S100 calcium binding protein B (S100β) (Cat No# ab52642 - Abcam, 1:600) and anti–antigen Kiel 67 (Ki67) (Cat No# GTX16667 - GeneTex Co., 1:200) overnight at 4 °C. After washing the sections, they were incubated for 20 min with 2^ry^ antibody HRP Envision kit (Cat No# K5007 - DAKO). After that, they were washed with Phosphate-buffered saline followed by 10 min incubation with diaminobenzidine (DAB). The last washing by Phosphate-buffered saline was followed by staining with hematoxylin, then dehydrated and cleared by xylene, then cover slipped for microscopic examination. At least 6 random non-overlapping fields were selected and scanned from cerebellar folia of each sample for the determination of SOX2 ++/S100β ++ mean reactive Bergmann (BG) glia cells as well as Ki67 ++ expressed cells as count per field. All data were recorded by using Leica Application module for tissue sections’ analysis attached to a Full HD microscopic imaging system (Leica Microsystems GmbH, Germany) operated by an examiner histologist.

### 2.8 Statistical analysis

The findings were presented as mean ± SD. The data were checked for normality using Shapiro-Wilks test and homogeneity of variance using Levene’s test. The differences between groups were evaluated using one-way ANOVA and a subsequent Tukey *post hoc* test. These analyses, along with the generation of figures, were executed by GraphPad Prism software (version 10). *P*-value <0.05 was deemed statistically significant.

## 3 Results

### 3.1 TELMI attenuates tremor severity

**FIGURE 1:**
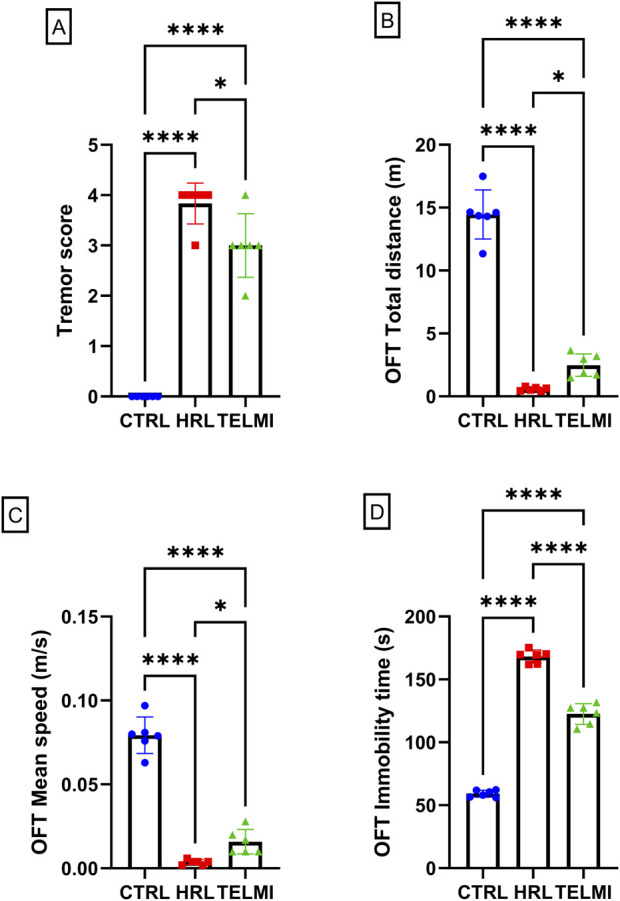
Impact of Telmisartan on tremor intensity and locomotor activity in ET rats. The vertical line in each bar represents the mean ± S.D. (for each parameter n = 6) **(A)** tremor score, **(B)** distance travelled, **(C)** mean speed and **(D)** immobility time. Statistical analysis was performed by one-way ANOVA followed by Tukey’s *post hoc* test, with the criterion for statistical significance: significant at P < 0.05. CTRL, Normal control; HRL, Harmaline; TELMI, Telmisartan; OFT, open field test; ns, Non-significant; *p < 0.05, **p < 0.01, ***p < 0.001, ****p < 0.0001.

According to Figure 1A, HRL animals showed high tremor scores *[F (2, 15) = 129.1, P < 0.0001, η2 = 0.94]*, indicating overall intensity of tremors. At the same time, rats pre-treated with TELMI exhibited mitigated tremors to reach 78.3% of the HRL group.

### 3.2 Effect of TELMI on spontaneous activity in HRL rats

Data in Figures 1B–D reveal that HRL-treated rats showed deficits in open field test manifested by a decrease in both total distance travelled *[F (2, 15) = 220.4, P < 0.0001, η2 = 0.96]* and mean speed *[F (2, 15) = 170.1, P < 0.0001, η2 = 0.95]* by 96% and 95.3%, respectively compared to CTRL rats. In a parallel manner, the immobility time of HRL rats *[F (2, 15) = 526.6, P < 0.0001, η2 = 0.98]* was prolonged to reach 284.1% of the CTRL group. On the other hand, prior administration of TELMI extended the total distance and speed each by more than 4-fold the value of HRL animals, while it succeeded in constricting the immobility time to 72.8% of HRL group.

### 3.3 Impact of TELMI on HRL-induced imbalance, muscle debility and gait disturbance

**FIGURE 2:**
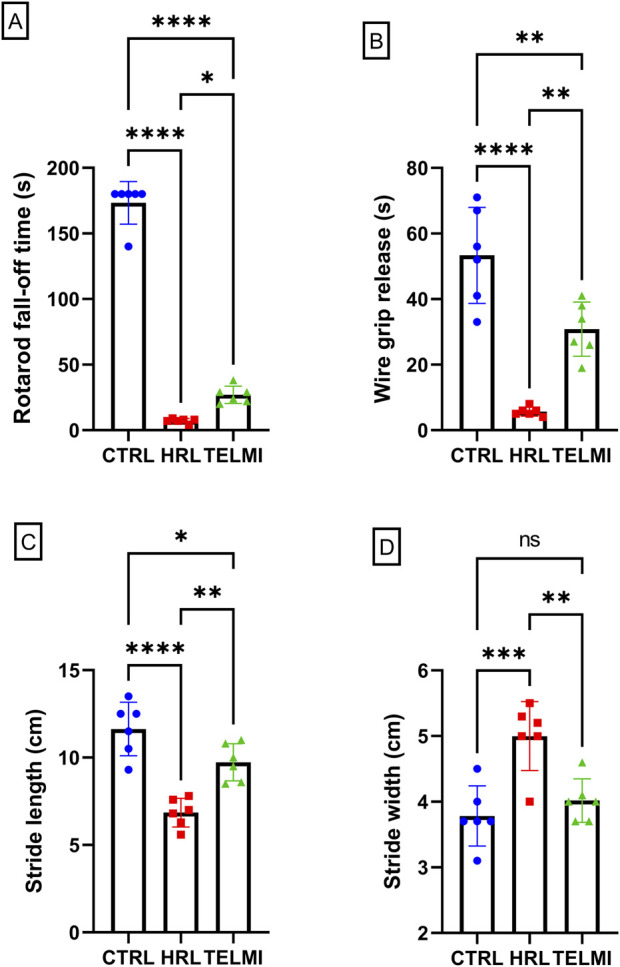
Telmisartan restores balance and motor functions in ET rats. The vertical line in each bar represents the mean ± S.D. (for each parameter n = 6) **(A)** rotarod fall-off time, **(B)** wire grip release time, **(C)** stride length and **(D)** stride width. Statistical analysis was performed by one-way ANOVA followed by Tukey’s *post hoc* test, with the criterion for statistical significance: significant at P < 0.05. CTRL: Normal control, HRL: Harmaline, TELMI: Telmisartan. ns; Non-significant, *p < 0.05, **p < 0.01, ***p < 0.001, ****p < 0.0001.

As illustrated in Figure 2, animals’ balance, muscle strength and gait coordination were severely disrupted in rotarod *[F (2, 15) = 475.1, P < 0.0001, η2 = 0.98]*, wire grip *[F (2, 15) = 35.9, P < 0.0001, η2 = 0.83]* and footprint analysis tests following HRL administration.

HRL-treated rats exhibited a major reduction in the time spent on the rotating rod by 95.8% compared to normal rats. This diminution was reversed after TELMI administration to increase the fall-off time by more than three folds of the HRL group (Figure 2A).

As seen in Figure 2B, HRL negatively affected the release time to show a tremendous reduction by 89.3%, with respect to CTRL animals. Fortunately, prior treatment with TELMI achieved a 5-fold delay in wire release time, compared to HRL rats.

In footprint analysis test, rats subjected to HRL presented a marked decline in stride length *[F (2, 15) = 25.15, P < 0.0001, η2 = 0.77]* to reach merely 59% of the CTRL rats along with an abnormal lengthening in stride width *[F (2, 15) = 12.61, P < 0.0001, η2 = 0.63]*, reaching 132.2% of normal animals. Meanwhile, TELMI pre-treated animals demonstrated an increase in stride length reaching 142% compared to HRL group (Figure 2C). This was accompanied by a retracted stride width in TELMI-treated animals to reach 80.4% of HRL group (Figure 2D).

### 3.4 Fluctuation in GABA and GLU levels in HRL rats

**FIGURE 3:**
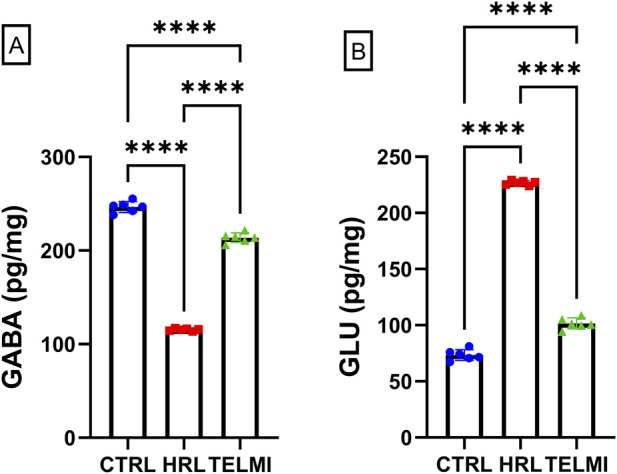
Neurotransmitters’ changes in cerebella of HRL and TELMI rats. The vertical line in each bar represents the mean ± S.D. (for each parameter n = 6) **(A)** GABA and **(B)** GLU. All evaluated parameters were performed using cerebella isolated from the extracted rat brains. Statistical analysis was performed by one-way ANOVA followed by Tukey’s *post hoc* test, with the criterion for statistical significance: significant at P < 0.05. GABA, gamma aminobutyric acid; GLU, glutamate; CTRL, Normal control; HRL, Harmaline; TELMI, Telmisartan; ns, Non-significant; *p < 0.05, **p < 0.01, ***p < 0.001, ****p < 0.0001.

Disturbances in motor performance are often accompanied by changes in levels of primary neurotransmitters. Data in Figure 3, shows that GABA level was severely reduced after ET induction by 53.2% compared to normal rats *[F (2, 15) = 1321, P < 0.0001, η2 = 0.99]*. On the other hand, HRL augmented GLU level by three folds compared to CTRL animals *[F (2, 15) = 2377, P < 0.0001, η2 = 0.99]*. Fortunately, TELMI restored GABA level to reach 185% compared to HRL group and reversed the increase in GLU to reach 44.8% the value in HRL animals.

### 3.5 Telmisartan curbed the inflammatory stream in HRL rats

**FIGURE 4:**
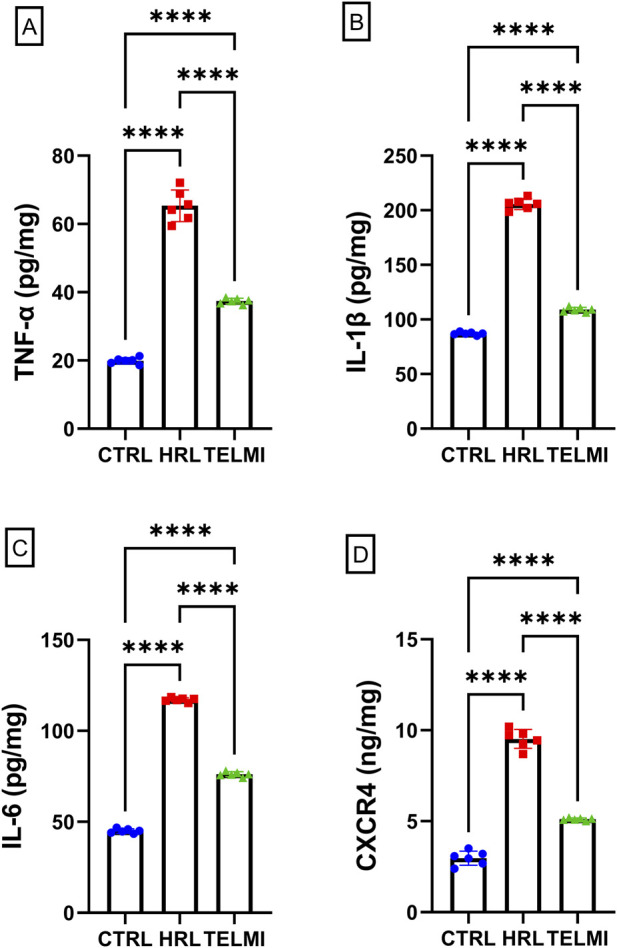
Telmisartan attenuates the neuroinflammatory outburst in rats’ cerebellar tissue. The vertical line in each bar represents the mean ± S.D. (for each parameter n = 6) **(A)** TNF-α, **(B)** IL-1β, **(C)** IL-6 and **(D)** CXCR4. All evaluated parameters were performed using cerebella isolated from the extracted rat brains. Statistical analysis was performed by one-way ANOVA followed by Tukey’s *post hoc* test, with the criterion for statistical significance: significant at P < 0.05. TNF-α, tumor necrosis factor alpha; IL-1β, interleukin one beta; IL-6, interleukin 6; CXCR4, chemokine receptor type 4; CTRL, Normal control; HRL, Harmaline; TELMI, Telmisartan; ns, Non-significant; *p < 0.05, **p < 0.01, ***p < 0.001, ****p < 0.0001.

As presented in Figure 4, rats injected with HRL showed a tremendous increment in inflammatory mediators, including TNF-α *[F (2, 15) = 408.1, P < 0.0001, η2 = 0.98]*, IL-1β [*F (2, 15) = 2307, P < 0.0001, η2 = 0.99]*, IL-6 *[F (2, 15) = 5177, P < 0.0001, η2 = 0.99]* and CXCR4 *[F (2, 15) = 477.2, P < 0.0001, η2 = 0.98]* to reach 328%, 236%, 260.3% and 320.8% respectively, compared to CTRL rats. At the same time, after TELMI treatment, the values were downgraded to 0.57, 0.53, 0.65 and 0.54 folds compared to HRL group.

### 3.6 Telmisartan’s impact on the ACE/Ang II/Ang 1-7 axis

**FIGURE 5:**
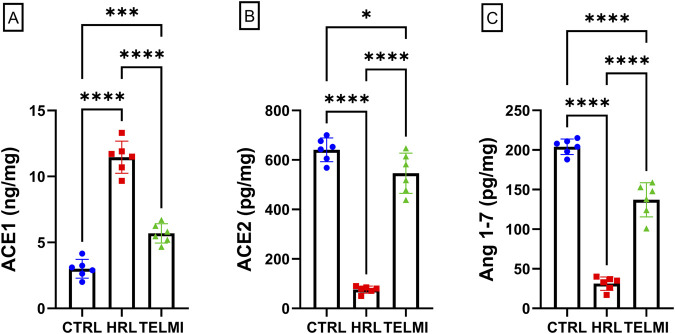
Role of cerebellar ACE1/2 and Ang 1-7 in ET. The vertical line in each bar represents the mean ± S.D. (for each parameter n = 6) **(A)** ACE1, **(B)** ACE2, **(C)** Ang 1–7. All evaluated parameters were performed using cerebella isolated from the extracted rat brains. Statistical analysis was performed by one-way ANOVA followed by Tukey’s *post hoc* test, with the criterion for statistical significance: significant at P < 0.05. ACE1: angiotensin converting enzyme 1, ACE2: angiotensin converting enzyme 2, Ang 1–7: angiotensin 1-7, CTRL: Normal control, HRL: Harmaline, TELMI: Telmisartan. ns; Non-significant, *p < 0.05, **p < 0.01, ***p < 0.001, ****p < 0.0001.

According to Figure 5, HRL rats presented a prominent expression of ACE1 enzyme to reach almost four folds the CTRL group *[F (2, 15) = 132.5, P < 0.0001, η2 = 0.94]*. Yet, the levels of ACE2 *[F (2, 15) = 180.4, P < 0.0001, η2 = 0.96]* and Ang 1–7 *[F (2, 15) = 215.7, P < 0.0001, η2 = 0.96]* were negatively affected, reaching 11.8% and 15.6%, respectively, compared to CTRL rats. Simultaneously, treatment with TELMI reduced the level of ACE1 to less than half the value in HRL group, while it succeeded in reinstating ACE2 and Ang 1-7 levels to attain 718% and 438% respectively, compared to HRL group.

### 3.7 Alterations in AT1, AT2 and MAS receptors after HRL and TELMI

Modulations in the ACE/Ang II/Ang 1-7 axis will be reiterated in the expression of the corresponding Ang II receptors.

**FIGURE 6:**
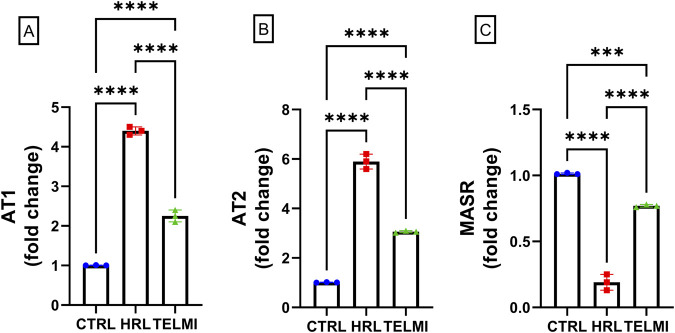
Inference of cerebellar AT receptors in ET pathogenesis. The vertical line in each bar represents the mean ± S.D. (for each parameter n = 3) **(A)** AT1, **(B)** AT2 and **(C)** MASR. All evaluated parameters were performed using cerebella isolated from the extracted rat brains. Statistical analysis was performed by one-way ANOVA followed by Tukey’s *post hoc* test, with the criterion for statistical significance: significant at P < 0.05. AT1, angiotensin II receptor type 1; AT2, angiotensin II receptor type 2; MASR, Mas receptor; CTRL, Normal control; HRL, Harmaline; TELMI, Telmisartan; ns, Non-significant; *p < 0.05, **p < 0.01, ***p < 0.001, ****p < 0.0001.

Data from Figure 6 reveal that rats subjected to HRL overexpressed both AT1 *[F (2, 6) = 816.7, P < 0.0001, η2 = 0.99]* and AT2 receptors *[F (2, 6) = 591.8, P < 0.0001, η2 = 0.99]*, reaching 438.7% and 584.1% respectively, compared to CTRL animals. Albeit the expression of MASR *[F (2, 6) = 431.3, P < 0.0001, η2 = 0.99]* was downregulated by 81.2% when compared to CTRL group. Conversely, AT1 and AT2 were normalized in TELMI animals to half the value in HRL rats for both types of receptors. Interestingly, MASR was expressed extensively after TELMI administration, reaching more than four folds the HRL group.

### 3.8 Impact of TELMI on p38 MAPK, progenitor and proliferative cell markers

**FIGURE 7:**
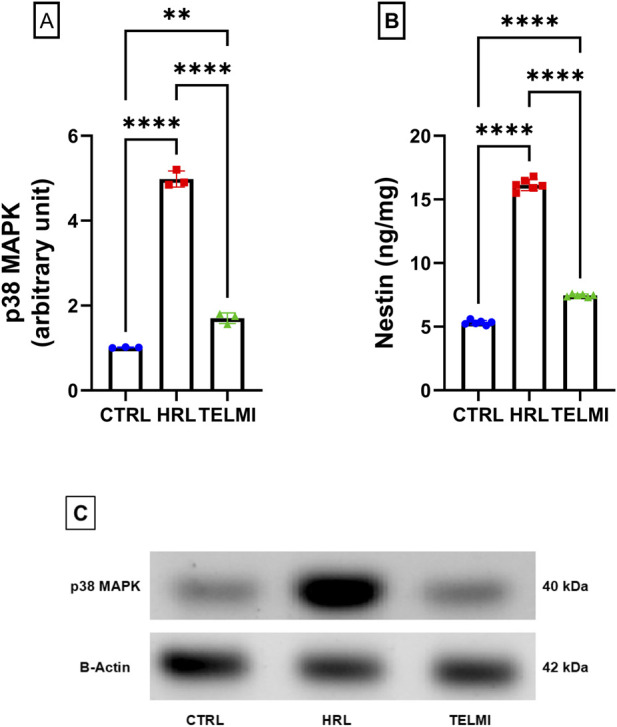
Influence of TELMI on cerebellar p38 MAPK pathway. The vertical line in each bar represents the mean ± S.D. **(A)** P38 MAPK (n = 3), **(B)** nestin (n = 6) and **(C)** Western blotting of p38 MAPK. All evaluated parameters were performed using cerebella isolated from the extracted rat brains. Statistical analysis was performed by one-way ANOVA followed by Tukey’s *post hoc* test, with the criterion for statistical significance: significant at P < 0.05. P38 MAPK: p38 mitogen-activated protein kinase, CTRL: Normal control, HRL: Harmaline, TELMI: Telmisartan. ns; Non-significant, *p < 0.05, **p < 0.01, ***p < 0.001, ****p < 0.0001.

The multipronged effects of MASR extend to p38 MAPK, which is involved in multiple mechanisms of cell cycle regulation. According to Figure 7, HRL prompted a sudden stimulation in p38 MAPK *[F (2, 6) = 780.1, P < 0.0001, η2 = 0.99]* and nestin *[F (2, 15) = 2402, P < 0.0001, η2 = 0.99]* to reach almost five and three folds the values in normal animals, respectively. Surprisingly, the application of TELMI mitigated the levels of these parameters to reach 34.2% and 46.2% for p38 MAPK and nestin, respectively, when compared to HRL group (Supplementary Figures).

**FIGURE 8:**
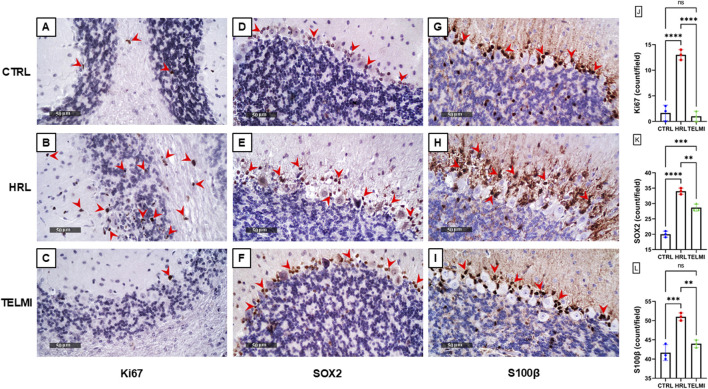
Effect of HRL and TELMI on progenitor and proliferative markers in cerebella. The vertical line in each bar represents the mean ± S.D. (for each parameter n = 3). Photomicrographs present **(A–C,J)** Ki67, **(D–F,K)** SOX2 and **(G–I,L)** S100β. The cerebellar folia were used for the determination of SOX2 ++/S100β ++ mean reactive Bergmann glia cells as well as Ki67 ++ expressed cells as count per field. Statistical analysis was performed by one-way ANOVA followed by Tukey’s *post hoc* test, with the criterion for statistical significance: significant at P < 0.05. Ki67, antigen Kiel 67; SOX2, SRY-box transcription factor 2; S100β, S100 calcium binding protein B; CTRL, Normal control; HRL, Harmaline; TELMI, Telmisartan; ns, Non-significant; *p < 0.05, **p < 0.01, ***p < 0.001, ****p < 0.0001.

A closer inspection of Figure 8 confirms the triggering action after HRL administration on proliferative and progenitor markers Ki67 *[F (2, 6) = 94.46, P < 0.0001, η2 = 0.96]*, SOX2 *[F (2, 6) = 134.8, P < 0.0001, η2 = 0.97]* and S100β *[F (2, 6) = 33.53, P = 0.0006, η2 = 0.91]*, as their expression in reactive BG glia cells leaped by 7.7, 1.7 and 1.2 folds respectively, compared to CTRL rats. Interestingly, TELMI rats displayed a lower expression of Ki67, SOX2 and S100β to 7.6%, 84.3% and 86.3% the values in HRL group respectively.

### 3.9 Cerebellar histopathological changes evoked by TELMI and HRL

**FIGURE 9:**
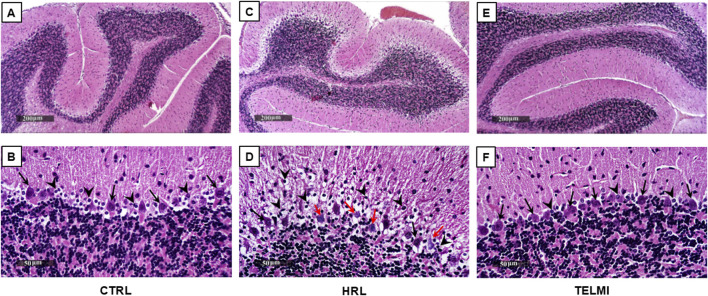
Photomicrographs representing light microscopic examination of cerebellar tissue from the assigned experimental groups using H&E stain, where **(A,B)** CTRL, **(C,D)** HRL **(E,F)** TELMI. CTRL: Normal control, HRL: Harmaline, TELMI: Telmisartan.

To further validate the behavioural and biochemical scrutiny, cerebellar tissue samples were examined. Normal CTRL samples showed well-preserved foliar architecture with an apparent intact outer molecular layer (Figure 9A). The Purkinje cell (PC) layer displays a single row of Purkinje neurons with prominent nuclei and well-defined cytoplasmic details (black arrow). Adjacent to these neurons, numerous supportive Bergmann (BG) glial cells are observed (arrowhead), (Figure 9B). The internal granular layer is densely populated with small, round granule cells exhibiting intact subcellular structures. Apparent intact intercellular brain matrix with no evidence of abnormal pathological alterations. On the other hand, HRL samples showed remarkable disorganization of PC layer as well as the adjacent inner molecular layer zone with extensive PC loss and degenerative and necrotic changes (red arrow), minimal display of apparent intact cells (black arrow), accompanied with remarkable increase of reactive glial cells infiltrates (arrowhead) (Figure 9D). Also, moderate to severe edema was observed in PC layer that extended to the outer molecular layer matrix (Figure 9C). At the same time TELMI samples showed evident neuroprotective efficacy with minimal neuronal degenerative changes (Figure 9E) and significantly higher records of apparent intact PCs (black arrow). Moreover, higher figures of supporting BG glial cells were evident (arrowhead) with minimal abnormal glial cells’ infiltrates (Figure 9F).

## 4 Discussion

In the current study, a novel prospective role for ACE2/Ang 1–7/MASR axis in HRL-induced ET model has been declared, opening up a new avenue for managing this neurological disorder. The current results prominently display the neuroprotective actions of TELMI, accentuated by the enhancement of ACE2/Ang 1–7/MASR trajectory, fostering suppression of p38 MAPK pathway with a consequential reduction in neuroinflammation. Additionally, the neuroprotective effect of TELMI was protracted to normalizing the levels of the persistent markers for neural stem cells; SOX2 and nestin, as well as the neurodegeneration marker; S100β and the proliferative marker, Ki67, in the cerebellum of HRL-injected rats. Such neurorestorative effects were further accompanied by correcting the GABA/GLU imbalance and improving the neurobehavioral functionality.

Disturbances in cerebellar function are associated with the development of various neuropsychiatric conditions, including Parkinson’s disease (PD), multiple sclerosis (MS), depression, schizophrenia, in addition to ET (Bareš et al., 2012). Indeed, ET might be the most common form of cerebellar degeneration, where this disruption elicits mild to moderate gait ataxia and other cognitive features, along with tremors. Although the patho-mechanisms of ET are still obscure, postmortem studies and clinical findings all recurrently fortify the fact that the cerebellum is tangled with ET pathogenesis (Klein et al., 2011). Mechanistically, it was conveyed that there is an alteration of the cerebellar timing function in patients with ET, displayed in the variability of rhythmic hand movements, and the impaired performance during predictive motor timing task. Such an observation can be explained by the fact that the cerebellum acts as an internal timing system; essential for coordinating the timing of movement and sensation (Bareš et al., 2012).

In the present work, treatment with TELMI tempered HRL-induced tremors as evident via enhancing locomotor activity and motor coordination evaluated by open field test and rotarod tests, as well as improving neuromuscular function and gait pattern estimated by wire grip and footprint tests. Such amelioration might be correlated with improving the histological injury in cerebellar tissues.

The current research addresses the question of to what extent the ACE2/Ang 1–7/MASR axis-dependent mechanism could contribute to the TELMI-induced protection of HRL-induced ET. The neuroprotective effect of Ang 1–7/MASR axis has been previously delineated across a plethora of pathological conditions. To illustrate, Ang 1–7 has proven advantageous effects in both ischemic and hemorrhagic stroke models, where Ang 1-7 administration has shown to dwindle the neurological deficits after the insult, attenuate cerebral inflammation, and ultimately improve the brain’s neurological function (Regenhardt et al., 2020). In another feature, emerging evidence implicates dysregulation of the brain ACE2/Ang 1–7/MASR axis in the pathogenesis of AD. In support of this, ACE2 activity was reported to be reduced by half in postmortem brain tissue from AD patients (Kehoe et al., 2016). This decline has been strongly associated with elevated Aß and phosphorylated tau levels; two key pathological hallmarks of AD pathophysiology. Besides, the brain Ang 1–7/MASR axis has been closely linked to attenuation of anxiety- and depressive-like behaviors in a transgenic rat model exhibiting these neurodisorders (Kangussu et al., 2013). These findings collectively support the notion that modulation of ACE2/Ang (1–7)/MASR axis can represent a novel candidate for the potential treatment of ET,via employing the beneficial effects on cognitive processes, and opposing the deleterious actions of the classical pressor arm of the RAS.

Herein, results demonstrated a significant enhancement in AT1 expression levels in the cerebellum of HRL-treated rats, along with a concomitant diminution in AT2 and MASR expression levels, a condition reversed followingTELMI treatment. Besides, TELMI administration induced an increase in Ang 1-7 levels, as well as, ACE2 level.

The RAS is known to be profoundly sensitive to both inflammatory and oxidative stressors. Undoubtedly, RAS blockade by ACE inhibitors (ACEIs) or angiotensin receptor blockers (ARBs) reduces AT1 receptor stimulation, leading to increased levels of ACE2. Consequently, levels of Ang1–7 become elevated following treatment with ACEIs or ARBs (Kriszta et al., 2021). In agreement, treatment with TELMI and/or 17-β estradiol reversed the induced inhibition of hippocampal MASR expression and increased the hippocampal protein expression of ACE2 in D-gal injected OVX rats (Abdelkader et al., 2020). In line, myocardial protein levels of ACE2 and MASR were upregulated after TELMI and olmesartan treatment, causing cardioprotective effects (Sukumaran et al., 2011). Besides, both TELMI and losartan significantly enhanced ACE2 mRNA levels, modulating the intrahepatic RAS favoring the ACE2/MASR axis over the ACE/AT1 axis, promoting beneficial metabolic effects in a model of high-fat diet-induced obesity (Murphy et al., 1995). Furthermore, the involvement of TELMI in other stress-responsive pathways affecting RAS is plausible. The mechanistic advantage of TELMI as a partial agonist of peroxisome proliferator-activated receptor-gamma (PPAR-γ) enhanced mitochondrial bioenergetics and cellular resilience against ROS and inflammatory outbursts. Several studies have proclaimed improvement in cognitive performance after TELMI and ascribed it to its role in restoring redox balance and anti-inflammatory capacity, supporting it as both a neurometabolic and neuroprotective modulator (Elma et al., 2026; Kishi et al., 2012; Kwon et al., 2021).

Simultaneously, TELMI caused inhibition of p38 MAPK phosphorylation, an effect previously elevated by HRL. In line with the current findings, administration of Ang 1–7 attenuated the Ang II-induced nociceptive behavior through a mechanism involving inhibition of p38 MAPK phosphorylation via spinal MASR in mice (Nemoto et al., 2014). Consistently, earlier research concluded that Ang 1–7/MAS axis provided protection in pancreatic cells through suppressing p38 MAPK signaling pathway in an *in vitro* model of acute pancreatitis (Yu et al., 2018). Together, these findings reinforce the concept that Ang 1–7/MASR pathway confers cellular protection, at least in part, through suppression of p38 MAPK signaling cascade.

Undeniably, p38 MAPK is particularly involved in neuroinflammation which has been identified as a central factor contributing to ET. Elevated levels of inflammatory markers have been revealed to be associated with the severity of ET and its cognitive and behavioral outcomes (Pirmoradi et al., 2025b). Markedly, eliciting a tremorgenic effect by HRL in the rat cerebellum triggered an elevation in the inflammatory status, as evidenced by the observed rise in CXCR4, TNF-α, IL-1β and IL-6. Nevertheless, the present findings revealed that TELMI treatment attenuated HRL-induced elevations in the inflammatory markers’ levels, bringing them closer to those observed in the control group.

Chemokine receptors play a critical role in the robust chemotactic response of inflammatory cells, such as neutrophils and lymphocytes, and in directing the migration of leukocytes from the circulation to sites of inflammation. CXCR4 is expressed by leukocytes and hematopoietic stem cells. The binding between CXCL12 and CXCR4 initiates downstream signaling pathways that are intricate in a myriad of pathological processes, including invasion of cancer and inflammatory disease (Tian et al., 2019). Preceding evidence also gives concrete proof that engagement of CXCL12 to its receptor; CXCR4, provokes intracellular signaling comprising the Erk/MAPK cascade, where it activates TNF-α transcription, secretion and receptor binding, stimulating cell death and activating nuclear factor kappa B (NF-κB) and Erk/MAPK that in turn translocate to the nucleus and activate interleukins’ expression (Han et al., 2001). In parallel, it was found that knockdown of CXCR4 inhibited the expression of IL-6 and TNF-α via suppressing MAPK and NF-κB signaling pathways in an acute lung injury model (Tian et al., 2019). Over and above, TNF-α initiates a second sequence of intracellular signaling events that leads to Ca^2+^ elevation, resulting in massive GLU release (Bezzi et al., 2001). Thus, our results extend to identify the complex chain of events that, starting from the activation of CXCR4 axis, leads to Ca^2+^-dependent GLU release. The current results coincide with Ma et al., who explained that CXCR4 knockout inhibits the degeneration of dopamine neurons, microglial and astrocyte activation, neuroinflammation, and BBB damages in PD mice (Ma et al., 2023).

Digging deeper to bring new insights into ET pathogenesis, we examined SOX2 expression through immunohistochemistry. SOX2 (Sex determining region of Y chromosome [SRY]-box transcription factor 2) is a founder member of the SOX family of transcription factors, which partake in critical functions in neurogenesis and maintenance of neural progenitor identity (Ding et al., 2012). As one of the earliest pluripotency transcription factors expressed in neural stem cells, SOX2 plays a key role in specifying early neural lineages and brain development, since it controls proliferation and differentiation of neural progenitor cells (NPCs) (Chen et al., 2019). Notably, SOX2 is consistently expressed in the cerebellar PC layer in adulthood (Mandalos et al., 2018). Prior evidence showed that increase in p38 activation promoted phosphorylation of SOX2, thus enhancing SOX2 stability, nuclear localization, and transcriptional activity in melanoma cells (Pietrobono et al., 2022). The current data showed that SOX2 was significantly upregulated in HRL-induced ET rat model, a condition that was ameliorated upon TELMI treatment. In harmony, SOX2-positive cells, in adult mice cerebellum, were shown to respond to physiological stimuli and increase in number upon local tissue damage. This fact portends the hypothesis that the SOX-positive BG population may be mobilizable, responding to damage and thus having the capability to be a local source of NPCs that could play a role in cerebellum repair (Salih et al., 2022).

In the context of deciphering potential functional roles of neurogenic biomolecules in the brain, nestin has been broadly considered as a reputed neuronal stem cell marker, typically associated with proliferative neuroprogenitors (Salih et al., 2022), and as one of SOX2-responsive target genes (Thiel, 2013). Nestin is a class IV intermediate filament (Zimmerman et al., 1994) that is vastly expressed in the developing CNS multipotent stem cells of the adult brain, under physiological conditions (Krishnasamy et al., 2017). However, its role in neuroinflammation and post-injury conditions remains significant and cannot be overlooked. Herein, there was a significant elevation in nestin level in HRL-induced tremor group, which was alleviated upon TELMI treatment. In parallel, a robust increase of nestin was previously linked to LPS-induced inflammation in the brain; a condition that was correlated with an increase in the GFAP-positive cells number (Krishnasamy et al., 2017). Previous studies also provide solid evidence that brain injuries and neurodegenerative disorders are intensely associated with inflammation, which is tightly related to neurogenesis (Darsalia et al., 2005), hence, an upsurge in neurogenesis has been observed in multiple models of neuroinflammation (Liu et al., 2009). In consistence, as regards the response of ependymal progenitors to spinal cord injury, nestin was found to be rapidly induced in cells of the spinal cord central canal by 2–4 days followed by decreased expression by 7–14 days post-injury. This was explained on the basis that spinal cord injury induces increased cell proliferation and differentiation primarily into macroglia or cells with nestin phenotype (Cizkova et al., 2009). Collectively, it could be concluded that under physiological conditions, nestin expression is restricted to NPCs in their respective niches, however, cerebellum injury induces its expression, where nestin-positive cells can differentiate to replace lost neurons. The upregulation of nestin could also prime BG cells to an activated stem cell-like state promoting proliferation and neurogenesis (Salih et al., 2022).

In the same milieu, S100β has been recognized as a reliable biomarker of active neural distress in a wide range of neurological disorders. Amplified levels of S100β have been detected in a variety of neuropathological conditions, including acute brain injury, neurodegenerative diseases; AD, PD, MS and psychiatric disorders such as schizophrenia and mood disorders. It is worth consideration that extracellular S100β has been shown to interact with surrounding cell types through the receptor for advanced glycation (RAGE) end products, initiating a complex signaling cascade that is linked to a series of pathological conditions, including neuroinflammation and neural injury (Michetti et al., 2019). As well, it was manifested that brain tissue injury and the proliferation of astrocytes lead to S100β overproduction. S100β is responsible for starting up a gliotic reaction by the release of pro-inflammatory mediators, including nitric oxide (NO) and cytokines from microglia and astrocytes, which have deleterious effects on neurons via enhancing reactive oxygen species production in neurons. Thus, increased S100β concentration is found to be proportional to the severity of the neural symptoms (Liu et al., 2011). The current data showed that there was a significant upregulation in S100β level upon administration of HRL. Significantly, TELMI succeeded in diminishing S100β in the cerebellar specimens. This comes consistent with another report that interposed S100β as a potential contributor to the development of PD, via provoking motor coordination impairment (Liu et al., 2011). In consistence, targeting S100β protein was elaborated to be effective in reducing induced epileptic seizures in rats following treatment with a traditional Chinese medicine herb; Uncaria rhynchophylla, which subsequently downregulated glial cell proliferation and attenuated neuronal death (Lin and Hsieh, 2011). It should be pointed out that S100β could induce oxidative stress by increasing lipid peroxidation of cell membrane, which in turn activates p38 MAPK, NF-κB and TNF-α (Esposito et al., 2006). Supporting data showed that several S100 proteins, extracellularly secreted from activated glial cells could trigger intracellular signaling cascades involving NF-κB and MAPKs, that drive the transcription of further pro-inflammatory cytokines and chemokines, intensifying the neuroinflammatory response (García-Domínguez, 2025). In addition, our findings suggest that Ki67 may be involved in the pathogenesis of neurodegeneration in ET. Mechanistically, p38 signaling cascade has been demonstrated to regulate the cell cycle and proliferation (Whitaker and Cook, 2021). As a cell proliferation marker, the heightened expression of Ki67 in HRL specimens indicates that these cells are not in the quiescent G0 phase. The results could be explained based on the relationships between the cell cycle and apoptosis, and the links between the cell cycle and cytoskeletal changes in neurodegeneration (Nagy et al., 1997). Formerly in AD, it was observed that cells arrested in G1 phase may eventually proceed to apoptotic cell death if they are unable to divide (Ucker DS, 1991).

Of note, the previous immunohistochemical results were demonstrated in the BG glia of the cerebellum. BG inflammation was demonstrated to be a major feature of several ataxic syndromes (Edamakanti et al., 2023). Moreover, BG cells are a prime example of highly diversified astrocytes that prompt diverse responses to support either tissue repair or promote CNS pathology in the context of trauma, infection, and neurodegenerative diseases (Federico Giovannoni, 2017). Light was shed on astrocyte heterogeneity and its potential neurotoxic activity which was proposed to participate in the pathogenesis of multiple neurologic diseases; AD, PD and MS (Colonna M and Brioschi, 2020). Generally, injury in the cerebellum triggers a complex mélange of compensatory regenerative events and regressive degenerative processes. Thus, the pathological alterations observed in the cerebellum of ET cases either epitomize successful regenerative attempts or denote aborted regenerative attempts, ultimately ending in degeneration (Louis and Faust, 2020).

Neurotransmitters’ disturbance, impacting the balance between excitatory and inhibitory neuronal activity in the tremor-related network, has also been conjectured in ET. This concept is consistent with the GABA hypothesis in ET, which entails a disturbance of the GABAergic system, especially in deep cerebellar neurons. Affirming the GABA hypothesis, it was found that there was a slight increase in the concentration of GLU and reduced levels of GABA, together with a decrease in glycine and serine in the cerebrospinal fluid and serum of ET patients (Salih et al., 2022). The present research addressed that the anti-tremor effects of TELMI could arise from its conspicuous effect on both GABAergic and glutamatergic axes. It has been suggested that altered levels of inhibitory GABA and excitatory GLU neurotransmitters, which occur in PC alterations, play a determining factor in tremorgenesis (Buijink et al., 2021). It has been elaborated that most cerebellar PCs release GABA, suppressing activity in the dentate nucleus and other cerebellar neurons to regulate and coordinate motor movements (Hebb, 2013). As well, concentrations of GABA_B_ receptors in the dentate nucleus, which mediate slow responses, were reported to be inversely associated with the duration of ET symptoms (Thier et al., 2011). In tandem, abnormal activation of climbing glutamatergic fibers, which arise from the inferior olive inducing synchronous firing of PCs of the cerebellar cortex, is presumed to be a “pacemaker” accountable for ET development (Fatemeh Rahimi Shourmasti et al., 2014). Such effect was confirmed by a study conducted by Akman et al., where agmatine, an endogenous neuromodulator, was conveyed to have anti-tremor effect in HRL model via blocking NMDA-type GLU receptors, which activate nitric oxide synthase, leading to NO production and enhancing glutamatergic activity (Akman et al., 2021). In parallel, riluzole, a GLU release inhibitor, was addressed to alleviate HRL-induced tremors and ataxia in rats through reducing the cerebellar GLU content and PC loss (Fatemeh Rahimi Shourmasti et al., 2014).

To explain, in pathological conditions, microglia release large amounts of TNF-α; as an important component of the neuroinflammatory response. TNF-α can potentiate glutamate-mediated cytotoxicity by two complementary mechanisms: indirectly, by inhibiting glutamate transport on astrocytes, and directly, by rapidly triggering the surface expression of AMPA and NMDA receptors, while decreasing inhibitory GABA receptors on neurons, culminating in a higher synaptic excitatory/inhibitory ratio (Olmos and Lladó, 2014).

One limitation of the study is that harmaline cannot be used chronically, as repeated administration leads to tolerance, besides, tremor involves multiple body regions in rodents (limbs, tail, trunk, head, whiskers), whereas ET in humans predominantly affects arms, hands, head, and voice. Additionally, ET is increasingly recognized as a heterogeneous syndrome with multiple possible etiologies. Hence, complementary chronic models-such as genetic tottering mice or 3xTg-AD mice—should be highlighted and used in parallel ET research. Future studies are recommended to incorporate these chronic models to assess long-term efficacy.

Modulating the RAS, particularly the angiotensin-(1–7)/MAS receptor axis, as a therapeutic intervention for ET remains at an early and largely speculative stage. The current clinical evidence involving RAS-modulating agents, such as ACEIs and ARBs, in motor or convulsive disorders is primarily derived from observational studies, retrospective analyses, or drug-repurposing investigations rather than well-designed randomized controlled trials. For instance, in a retrospective study, ACEIs/ARBs showed potential reduction in dyskinesia in PD; however, these findings are preliminary and require confirmation (Contaldi et al., 2021). Similarly, cohort studies have reported an association between ARB use—particularly Telmisartan—and a reduced incidence of epilepsy, though these findings remain associative and warrant validation in controlled trials (Contaldi et al., 2021; Doege et al., 2022). Additional observational evidence also suggests possible benefits of ARBs on non-motor symptoms such as anxiety in PD (Bordet et al., 2025). In contrast, population-based studies have not demonstrated a protective effect of antihypertensive medications on incident ET or related neurodegenerative disorders (Louis et al., 2009), although ARBs were not specifically evaluated as a distinct mechanistic class in that context. Notably, as of April 2026, no phase II or III clinical trials have directly investigated RAS-targeting approaches in ET or other motor disorders. Therefore, targeting of the RAS—particularly via the angiotensin-(1–7)/MAS receptor pathway—would be promising but requires further preclinical and clinical investigation.

Taken as a whole, the current study advocates a novel approach that underscores a potential for improving ET treatment. Additionally, it provides a comprehensive analysis of the molecular mechanisms involved in Ang 1–7/MASR pathway in an attempt to obtain a more in-depth understanding of how activating this pathway may contribute in dwindling of this neurological disorder and ultimately enhance its treatment strategies. Nevertheless, a definitive causal confirmation advocates additional studies addressing the role of telmisartan in PPAR-γ activation and its implication in ET as well as employing selective RAS inhibitors, MAS receptor antagonists, or genetic approaches.

## Data Availability

The original contributions presented in the study are included in the article/Supplementary Material, further inquiries can be directed to the corresponding author.

## Glossary

Aβ: amyloid-beta
ACE1: angiotensin converting enzyme 1
ACE2: angiotensin converting enzyme 2
ACEIs: ACE inhibitors
AD: Alzheimer’s disease
Ang 1–7: angiotensin 1–7
ANOVA: analysis of variance
ARBs: angiotensin receptor blockers
AT1: angiotensin II type 1 receptor
AT2: angiotensin II type 2 receptor
BG: Bergmann
CNS: central nervous system
CXCR4: chemokine receptor type 4
DAB: diaminobenzidine
DMSO: dimethyl sulfoxide
ERK: extracellular signal-regulated kinase
ET: essential tremor
GABA: gamma aminobutyric acid
GLU: glutamate
H&E: Hematoxylin and Eosin
HRL: harmaline
HRP: horseradish peroxidase
IL-1β: interleukin-1 beta
IL-6: interleukin-6
i.p: intraperitoneally
Ki67: antigen Kiel 67
MASR: Mas receptor
MS: multiple sclerosis
NF-κB: nuclear factor kappa B
NO: nitric oxide
NPCs: neural progenitor cells
OFT: open field test
p38 MAPK: p38 mitogen-activated protein kinase
PBS: phosphate buffer saline
PCs: Purkinje cells
PD: Parkinson’s disease
p.o.: orally
PPAR-γ: peroxisome proliferator-activated receptor-gamma
RAS: Renin-angiotensin system
S100β: S100 calcium binding protein B
SD: standard deviation
SOX2: SRY-box transcription factor 2
SDS: sodium dodecyl sulfate
TELMI: telmisartan
TNF-α: tumor necrosis factor alpha
**ηp** ^**2**^: effect size partial eta squared
