# Supplementary material for: Neuroprotective effects of telmisartan in a harmaline-induced model of essential tremor: modulation of the renin-angiotensin system and inflammatory pathways
Source: Front Pharmacol. 2026 May 26;17:1810499. doi: 10.3389/fphar.2026.1810499 (PMC13266293; doi:10.3389/fphar.2026.1810499)

Neuroprotective Effects of Telmisartan in a Harmaline-Induced Model of Essential Tremor: Mdulation of the Renin-Angiotensin System and Inflammatory Pathways

Sama M. Farrag[5], Amr M. Emam[5], Ahmed S. Kamel[1,4], Muhammed A. Saad[2,1*], Mona A. Kortam[2] , Noha H. Sayed[2], Nevine Fathy[2]

1 Department of Pharmacology and Toxicology, Faculty of Pharmacy, Cairo University, Cairo, Egypt.

2 Department of Pharmaceutical Sciences, College of Pharmacy, Gulf Medical University, Ajman, 4184, United Arab Emirates.

3 Department of Biochemistry, Faculty of Pharmacy, Cairo University, Cairo, Egypt

4 Department of Pharmacology and Toxicology, Faculty of Pharmacy and Drug Technology, Egyptian Chinese University, Gesr El Suez street, PO 11786 Cairo, Egypt

5 Department of Pharmacology and Toxicology, Faculty of Pharmaceutical Sciences and Drug Manufacturing, Misr University for Science and Technology (MUST), 6th of October City, Giza, Egypt

Supplementary Figure S1:

3 repeats of full gel of **p38 MAPK** Western blot image.

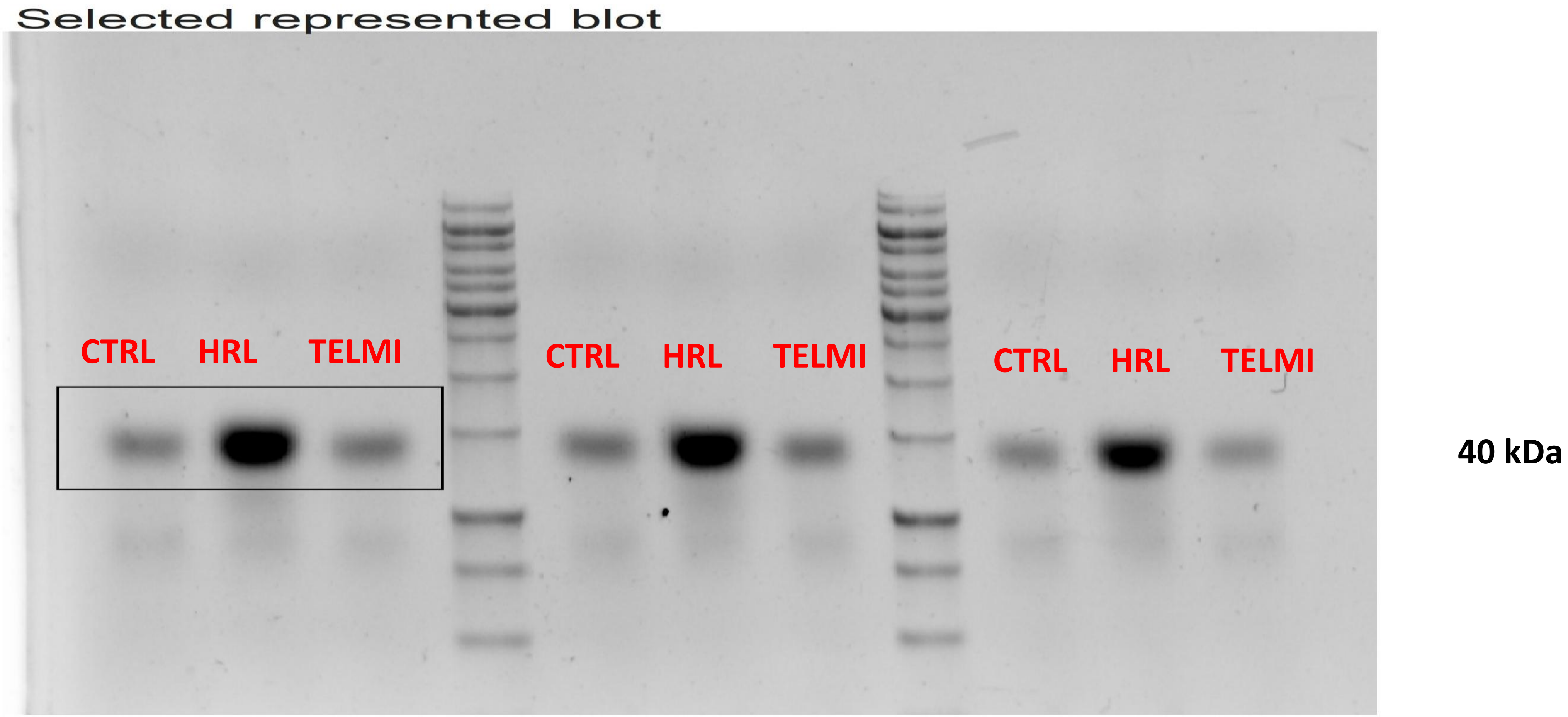

Supplementary Figure:
3 repeats of full gel of **β-actin** Western blot image.

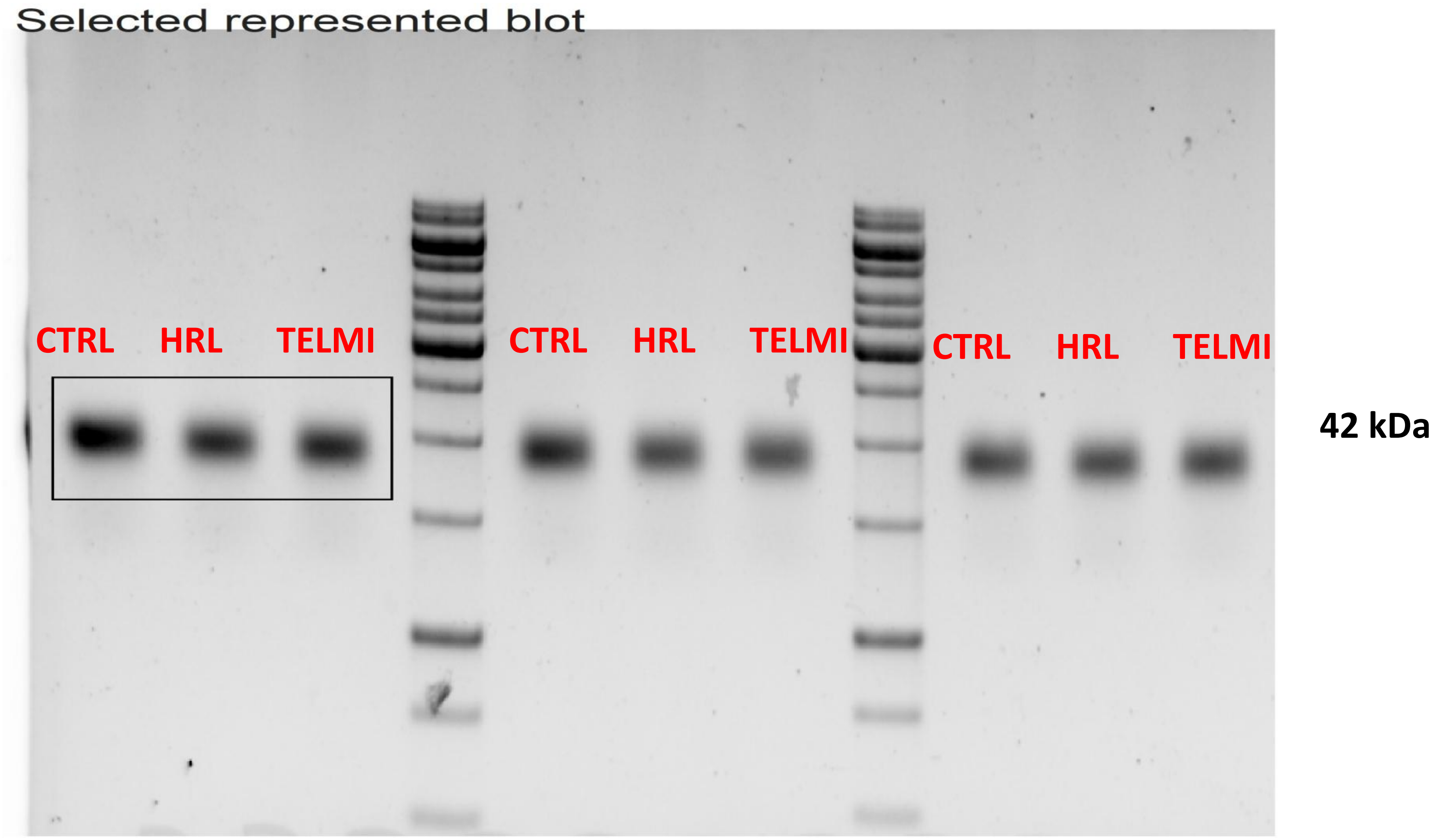

Supplement: Supplementary file 1 [file DataSheet1.pdf]
